# Supplementary material for: Deep learning-based classification of breast cancer molecular subtypes from H&E whole-slide images
Source: J Pathol Inform. 2024 Nov 17;16:100410. doi: 10.1016/j.jpi.2024.100410 (PMC11667687; doi:10.1016/j.jpi.2024.100410)
Supplement: Appendix A — Supplementary figures [file mmc1.pdf]

Supplementary Material

Figures

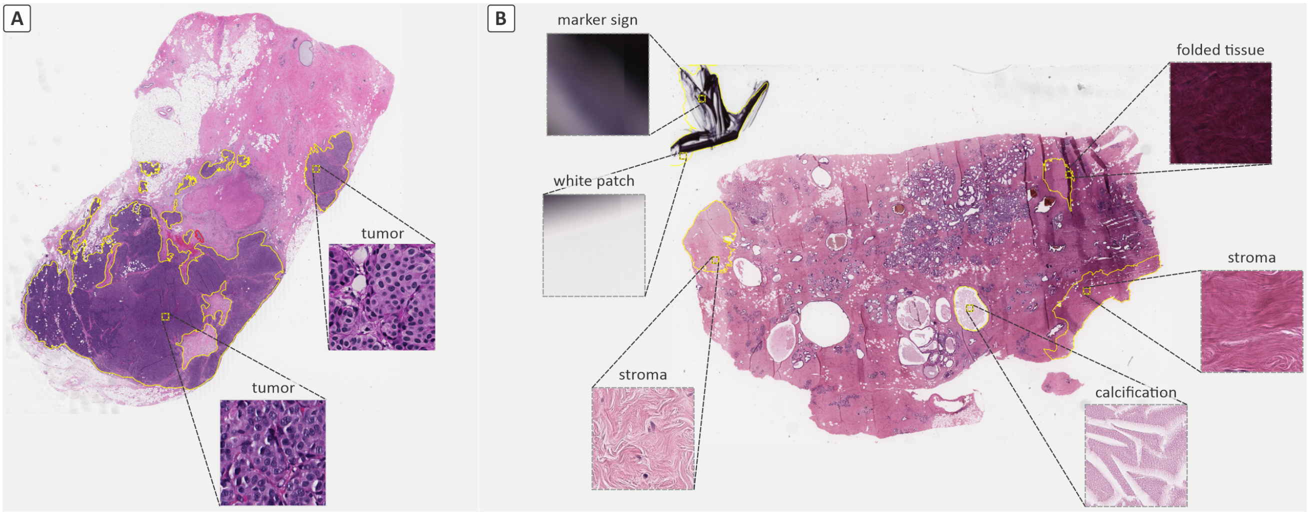

Figure 1: Extracting image tiles from breast H&E WSIs. (A) Tiling the annotated tumor regions from a WSI in the TCGA-BRCA dataset. (B) Examples of non-tumor tiles extracted from WSIs, including marker signs, normal tissues, folded tissue artifacts, and white areas of background.

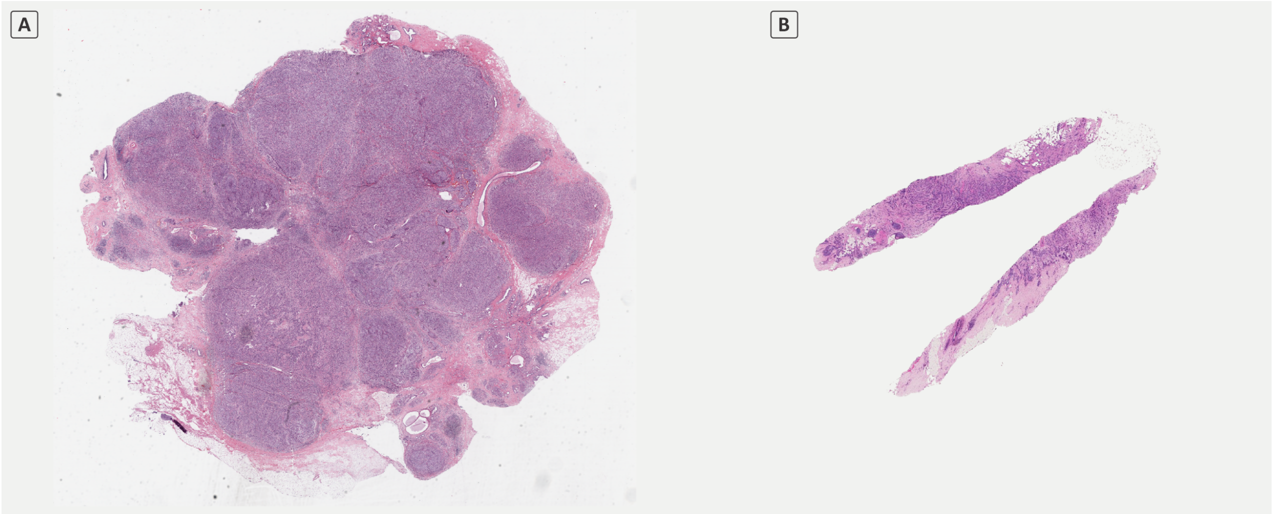

Figure 2: Comparison of harvested tissue between resection and biopsy: (A) a WSI from the TCGA-BRCA dataset, showing a breast tissue resection with an area of 2.32 cm^2, and (B) a WSI from the HER2-Warwick dataset, illustrating a breast biopsy with an area of 0.26 cm^2.

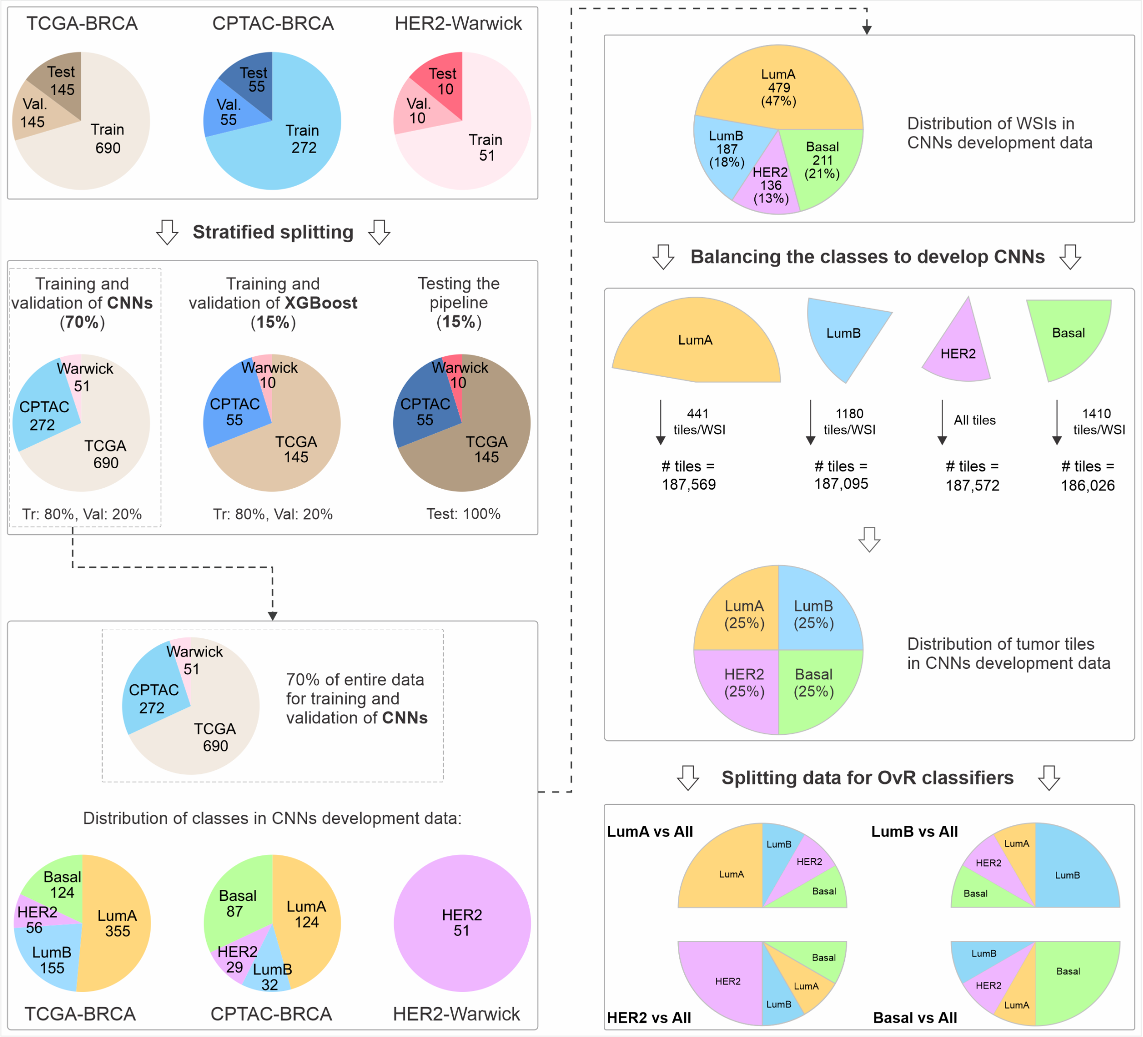

Figure 3: Illustration of data partitioning for training four binary One-vs-Rest classifiers.

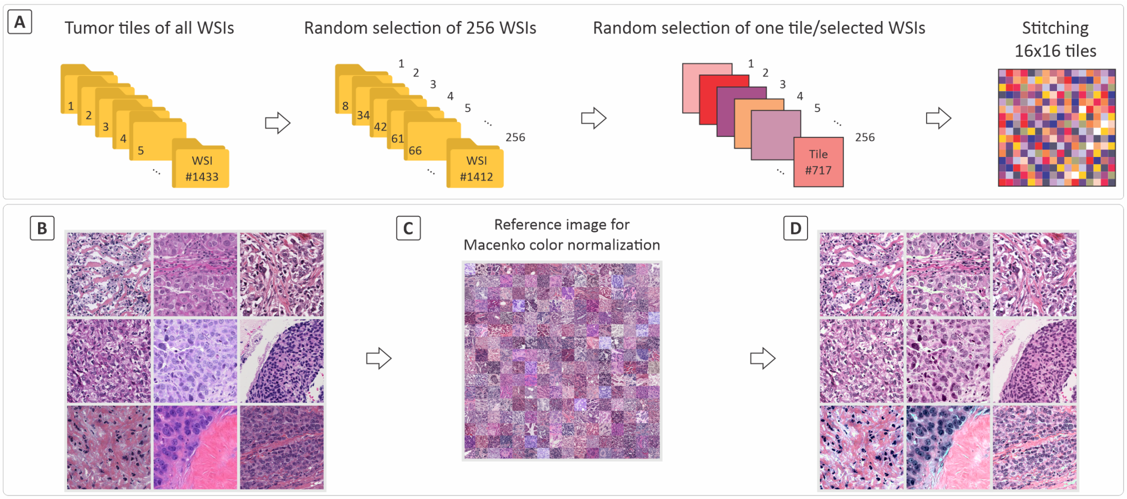

Figure 4: Color normalization of the tiles extracted from three different datasets: (A) Procedure of creating the reference image. (B) Examples of original tiles showcasing inherent color variations in images. (C) The reference image, made out of 256 tiles from 256 randomly selected WSIs. (D) Normalized images demonstrating reduced color variation in image tiles.

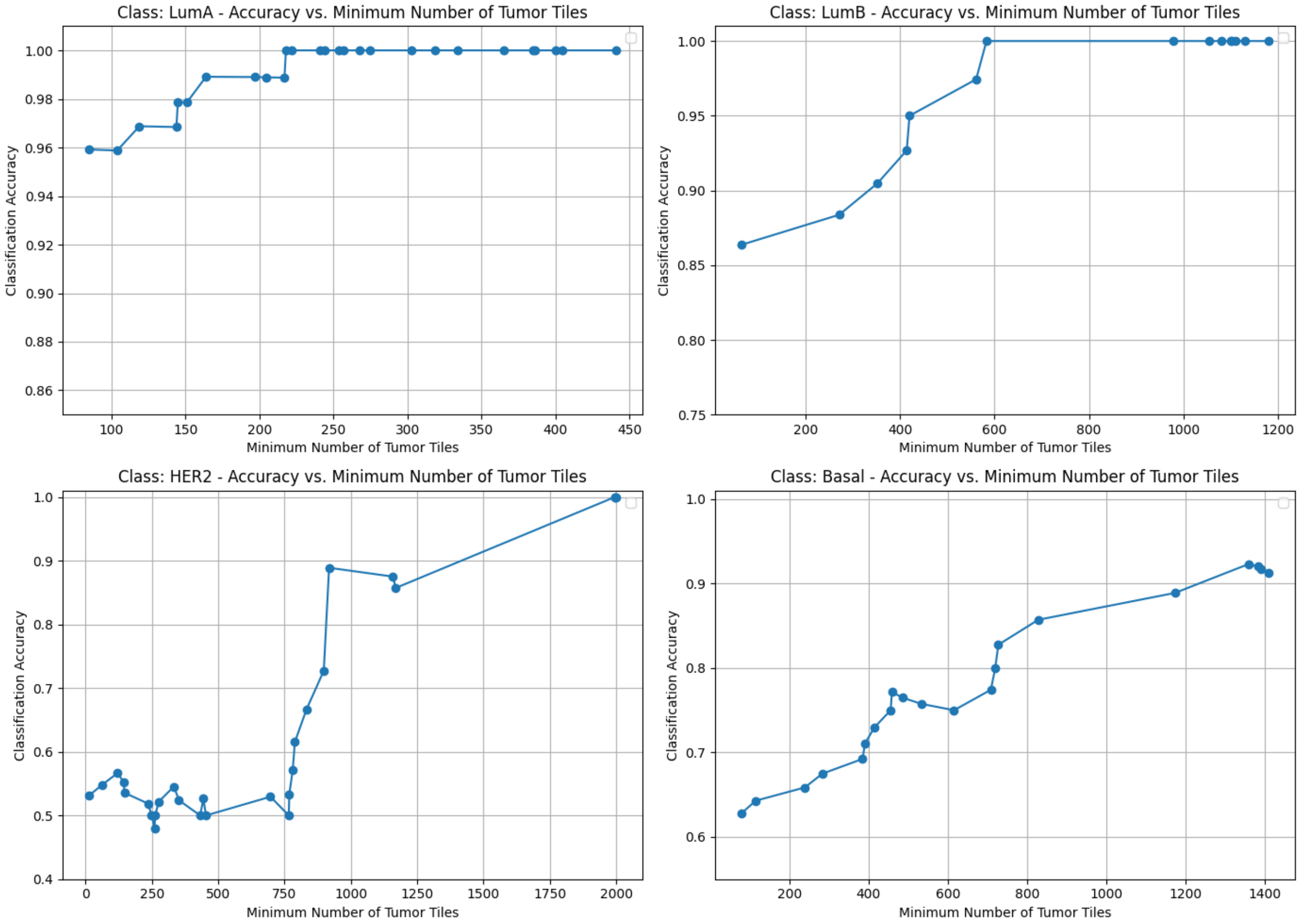

Figure 5: Accuracy of the breast cancer molecular subtype classifier as a function of the number of tumor tiles, showing an overall positive trend across all four classes.
